# Post-Recovery Relapse of Children Treated with a Simplified, Combined Nutrition Treatment Protocol in Mali: A Prospective Cohort Study

**DOI:** 10.3390/nu15112636

**Published:** 2023-06-05

**Authors:** Suvi T. Kangas, Issa Niamanto Coulibaly, Zachary Tausanovitch, Bareye Ouologuem, Bethany Marron, Elizabeth Radin, Christian Ritz, Salimou Dembele, Césaire T. Ouédraogo, Jeanette Bailey

**Affiliations:** 1 International Rescue Committee, New York, NY, USA; 2 International Rescue Committee, Bamako, Mali; 3 Nutrition Division, Ministry of Health, Bamako, Mali; 4 National Institute of Public Health, Copenhagen, Denmark

**Keywords:** acute malnutrition, combined protocol, community-based management of acute malnutrition, Mali, mid-upper-arm circumference, moderate acute malnutrition, ready-to-use therapeutic food, relapse, severe acute malnutrition, simplified protocol

## Abstract

The present study aimed to determine the 6-month incidence of relapse and associated factors among children who recovered from acute malnutrition (AM) following mid-upper arm circumference (MUAC)-based simplified combined treatment using the ComPAS protocol. A prospective cohort of 420 children who had reached a MUAC ≥ 125 mm for two consecutive measures was monitored between December 2020 and October 2021. Children were seen at home fortnightly for 6 months. The overall 6-month cumulative incidence of relapse [95%CI] into MUAC < 125 mm and/or edema was 26.1% [21.7; 30.8] and 1.7% [0.6; 3.6] to MUAC < 115 mm and/or edema. Relapse was similar among children initially admitted to treatment with a MUAC < 115 mm and/or oedema and among those with a MUAC ≥ 115 mm but <125 mm. Relapse was predicted by lower anthropometry both at admission to and discharge from treatment, and a higher number of illness episodes per month of follow-up. Having a vaccination card, using an improved water source, having agriculture as the main source of income, and increases in caregiver workload during follow-up all protected from relapse. Children discharged as recovered from AM remain at risk of relapsing into AM. To achieve reduction in relapse, recovery criteria may need to be revised and post-discharge strategies tested.

## 1. Introduction

Acute malnutrition is a condition that affects more than 45 million children under the age of five at any time [1] and requires specific treatment to ensure patient survival [2,3,4]. Most patients can be managed as outpatients with regular monitoring to ensure adequate evolution of nutritional status [2,3]. Treatment involves the provision of a weekly home ration of ready-to-use foods (RUF) and nutritional education [3,5]. For severe cases [6], the RUF dose aims at covering all the nutritional needs of the recovering child while for moderate cases the RUF dose supplements the regular diet. With treatment following the WHO recommended protocol [2,4], the cure rate of even severely malnourished children is high and few patients die [7].

Nonetheless, in recent years, questions have arisen about the long-term survival and health of children treated for malnutrition [8]. This is linked to observations of high relapse rates in children recovered from an episode of malnutrition [8]. However, few reliable data exist on the occurrence of relapses and the factors influencing relapses are poorly understood [8,9,10].

At the same time, despite the existence of an effective protocol for treating malnutrition at the level of health structures [2,3,4], current strategies have not been able to reach all malnourished children. In 2020, UNICEF reported that 5 million children accessed treatment [11], which translates to a <20% coverage of malnutrition care globally [12,13]. To support the scaling of treatment, simplified, combined approaches to detect and treat malnutrition have emerged proposing different adaptations to the treatment protocol [14].

In Mali, since 2018, the Ministry of Health, in collaboration with UNICEF, IRC, and several donors, has been implementing an operational pilot on simplified approaches for the treatment of acute malnutrition in children between 6 months and 5 years of age. Three modifications to the existing protocol have been put in place: (1) the management of acute malnutrition with a simplified and combined protocol [15,16]; (2) treatment delivery at both the health facility level by formal healthcare staff and at the community level by community health workers (CHWs) [17,18,19,20]; (3) training women to screen children for malnutrition using the “Family MUAC” approach [21,22,23,24]. The pilot has been implemented throughout the health district of Nara in the Koulikoro region including 35 health centers and 38 CHW sites and rural maternities, and the programmatic results of this approach are promising [25]. By December 2021, out of the 27,800 children admitted to simplified treatment, the recovery percent was 92%, the defaulter percent was 7%, and the death percent was 0.07% [25].

However, the relapse rate of these children after recovery is unknown. Since the protocol uses a lower dose of ready-to-use therapeutic food (RUTF) among certain sub-groups compared to the standard protocol and does not monitor other anthropometric indices than mid-upper arm circumference (MUAC), it is important to assess the performance of this protocol in maintaining recovery beyond treatment phase. In a follow-up study from the ComPAS trial in Kenya, no differences were found in the proportion of relapses between children treated with the simplified versus standard protocol 4 months after discharge from treatment [26]. In general, more research on relapse post-discharge has been recommended both by the World Health Organisation (WHO) [2] as well as the Council of Research and Technical Advice on Acute malnutrition (CORTASAM) [27].

This study’s aims were twofold: (1) to describe the incidence of relapse in the 6 months after successful treatment with the simplified and combined protocol, and (2) to investigate the factors associated with relapse.

## 2. Materials and Methods

### 2.1. Study Design and Population

This was a prospective cohort study enrolling children that had recovered from malnutrition following treatment with the simplified, combined protocol as per the treatment protocol used in the ComPAS study [15,16]. The study was nested within an on-going effectiveness study implementing the simplified and combined protocol at the scale of a full district including 35 health areas. As the district was large, a random sample of 10 health areas was selected using weights proportional to number of children treated per area to participate in this relapse study.

The 10 health areas included 10 health centers and 18 community health sites attached to the health centers. Caregivers of children who were treated for malnutrition using the simplified combined protocol in the selected health areas, and who achieved recovery defined as a MUAC ≥ 125 mm and no edema for two consecutive measurements, were invited to participate in the relapse study. Eligibility criteria included residing in the villages formally covered by the health area and accessible security wise for the study enumerators, the caregiver not planning on travelling in the next 6 months, and the child not having already participated in the study.

### 2.2. Study Setting and Treatment in Place

The study was set in the health district of Nara in southwestern Mali. The region is predominantly arid land [28], and the population subsists on small-scale farming and herding [29]. Food security in the region was estimated as stable in 2020 and 2021 [30]. However, access to healthcare remains an issue, with 29% of the population in the district living over 15 km away from basic services [31]. In the region, only 48% of children 12–24 months of age are estimated to be fully vaccinated [32], and malaria prevalence is 22% among children under 5 years of age [32]. Acute malnutrition prevalence as measured by a weight-for height z-score (WHZ) <−2 was estimated at 7.6% in 2019 [33].

The simplified, combined treatment protocol that was in place in the district during the study implementation is based on the protocol studied in the ComPAS trial [15,16] and included (1) admitting children to treatment on the basis of their MUAC measure (<125 mm) or presence of bilateral edema, (2) treating children with MUAC <115 mm and/or edema with 2 daily sachets of RUTF and children with a MUAC between 115 mm and 124 mm with 1 daily sachet of RUTF, (3) transitioning children admitted with MUAC < 115 mm to receiving 1 sachet per day after 2 weeks with a MUAC ≥ 115 mm, and (4) discharging children after 2 consecutive measures of MUAC ≥ 125 mm and absence of edema since 2 weeks. During treatment, all children were followed up weekly at the treatment site, and a discharge ratio of 7 sachets of RUTF was provided to all children at recovery.

### 2.3. Data Collection and Procedures

Each study area had one enumerator in charge of enrolment and a follow-up of participants. In order to enroll participants upon recovery from treatment, study enumerators were present at the treatment sites on treatment days and kept close contact with health agents both at the health center and community health level. Caregivers of children exiting treatment as recovered at the 10 participating health areas were approached by the enumerator to explain the study and request for consent. Follow-up was done on a fortnightly schedule through home visits. If the child and caregiver were absent from the household for 2 consecutive visits (1 full month), they were declared defaulted, and follow-up was ended.

All data were collected on tablets using the CommCare application. Upon enrollment, data concerning the child’s treatment path was captured: anthropometric measurements upon admission and discharge from treatment. During the first home visit, data on the characteristics of the household, the health environment, and food security were collected. All home visits included weight and MUAC measurements and questions on morbidity and childcaring and feeding practices. In addition, height measurement, changes to food security, and caregiver workload and income were collected monthly. To note, children treated at the CHW sites did not have their height measured at admission nor at discharge from treatment.

### 2.4. Outcomes and Sample Size

As per the CORTASAM recommendation to apply a relapse definition that aligns with the programs admission criteria [9], a MUAC-based definition of relapse was applied [25]. Relapse into acute malnutrition was defined as a MUAC < 125 mm or edema at any point post-recovery, as measured by the study enumerators upon the fortnightly home visits. All relapses were referred back to care to the closest treatment site.

The expected number of children needed for the present study was calculated to be 417, on the basis of the assumptions of a relapse percent during the 6 months of 40%, a loss to follow-up rate of 10%, margin of error of 6%, a 5% significance level, a 1.5 design effect, and a 95% confidence level. Dividing 417 children into 10 health areas meant recruiting 42 children per health area.

### 2.5. Predictors

Characteristics studied as potential predictors of relapse pertain to six variable groups, namely, (1) demographics and characteristics at admission to malnutrition treatment, (2) characteristics at discharge recovered from treatment, (3) socio-economic status, (4) dietary habits, (5) morbidity during post-discharge follow-up, and (6) changes at the household level during follow-up.

Demographics included sex and age at admission to treatment. Admission characteristics included WHZ, weight-for-age z-score (WAZ), height-for-age z-score (HAZ), and MUAC as continuous variables and as categorical ones looking at WHZ < −3, WAZ < −3, HAZ < −3, and MUAC < 115 mm at admission to treatment. In addition, admission site (formal health center versus CHW site) was studied. Discharge characteristics included WHZ, WAZ, HAZ, and MUAC as continuous and categorical ones looking at WHZ < −2, WAZ < −3, HAZ < −3, and MUAC < 130 mm at discharge recovered. Availability of a vaccination card and information on whether vaccines were up to date was collected at discharge, and length of stay in treatment was calculated. Socio-economic characteristics included household size, number of children under 5 years of age, agriculture as the main source of income, caregiver occupied as housewife, use of improved water source, treatment of drinking water, use of latrines, dirty appearance of the child, and household hunger. Household hunger was calculated on the basis of the Household Hunger Scale (HHS) [34]. Dietary habits included breastfeeding, Minimum Dietary Diversity (MDD), Minimum Meal Frequency (MMF), and Minimum Dietary Adequacy (MDA). MDD, MMF, and MDA were calculated as per the UNICEF 2020 guidelines [35], respecting the age categories and only for children <24 months of age. Morbidity variables studied included number of days with illness, number of days with illness per month, and having sought formal treatment upon illness. Changes in characteristics studied included change in household income, caregiver workload, and household hunger.

### 2.6. Data Management and Analysis

Baseline characteristics of the study population are summarized as percent (n) or mean ± SD or median (IQR) if non-normally distributed. Following the Council of Research and Technical Advice on Acute Malnutrition (CORTASAM) guidance published in 2020, the prevalence, cumulative incidences, and incidence rate (per 100 child-months) of relapse with 95% confidence intervals (95%CI) were calculated [9].

The cumulative incidence was defined as the number of children who relapse from 0 to 6 months after their discharge as recovered out of (a) the total number of children enrolled in the study and (b) number of children that continued through the 6 months without defaulting, consent withdrawal, or death. The incidence rate was the total number of episodes of relapse per 100 child-months. Monthly person time was calculated on the basis of the exit month for the child so that a child that exited the study at 4 months contributed 4 person months to the study. To describe the incidence rates according to the children’s nutritional status at admission to treatment, the incidence rates were also stratified according to admission category. 

Missing height data (due to random omission by health workers or systematic omission by CHWs as they were not taking height measures) was imputed on the basis of weight, age, timepoint in treatment, and any previous or later height measurements, in order to enable the analysis of association of relapse with height dependent z-scores HAZ and WHZ. Anthropometric z-score outliers (z-scores < −7 or >7 SD) were omitted.

Shared frailty models were fitted to quantify effects of predictors on time to recovery using health facilities as random effects and by assuming a Weibull distribution for the baseline hazard function. Both unadjusted and models adjusted for sex and age at enrolment were fitted. A *p*-value below 0.05 was used to declare statistical significance. All analyses were performed and plots were produced using STATA 15 (StataCorp, College Station, TX, USA). In order to estimate the heterogeneity of relapse rates between health areas (and by extension enumerators), the intra-cluster correlation coefficient (ICC) was estimated using a linear mixed model with relapse as outcome and clusters as random effects.

### 2.7. Ethics

This study was granted ethical approval by the International Rescue Committee Institutional Review Board (N° H1.00.026) and obtained approval for implementation by the Mali Ministry of Health and Social Development General Direction of Health and Public Hygiene (N° 1892/MSDS/DGSHP). Caregivers provided written informed consent prior to enrolment for all children included in the study. No compensation was provided for participation.

## 3. Results

### 3.1. Inclusion

**Figure 1:**
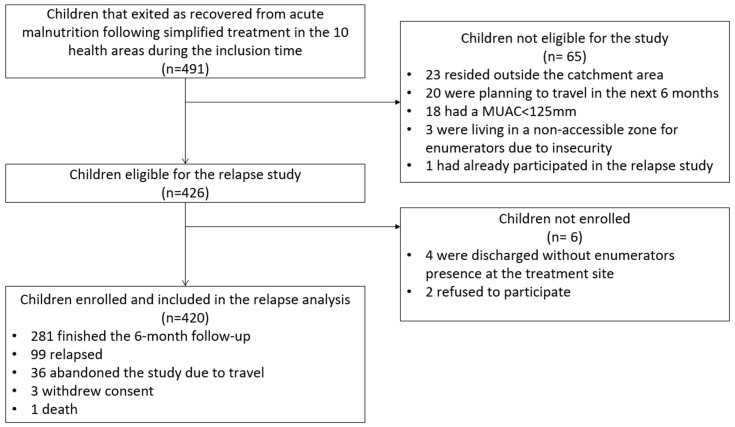
Patient flow chart for inclusion into the relapse study and analysis.

The enrolment of children started on 10 December 2020 and ended on 30 April 2021. Out of a total of 491 children that exited treatment as recovered during the inclusion period in the catchment area of the 10 participating health areas, 65 were excluded due to not meeting eligibility criteria. Out of the 426 eligible, 6 were not included due to either refusal or enumerator not being present at the treatment site upon discharge. A total of 420 children were included in the study (Figure 1).

### 3.2. Data Quality

A total of 24 anthropometric data points were excluded, including 2 admission WHZ measures, 2 admission HAZ measures, 6 discharge WAZ measures, 7 discharge WHZ measures, and 7 discharge HAZ measures due to being >7 or <−7 SD.

### 3.3. Participant Characteristics

Characteristics of children included in the study are described in Table 1. Children were on average 13 months old at admission to treatment, 48% were male, and their mean WHZ was −1.2, mean WAZ −2.0 and mean HAZ −2.1 at discharge from treatment. Only three children (1%) had a WHZ < −3 at discharge from treatment. Most children (74%) had been admitted to treatment with a MUAC between 115 mm and 124 mm, and 65% had been treated at the formal health center level as opposed to CHW sites. Up to 81% of caregivers reported agriculture as the primary source of income, and 72% declared not having other activities outside of being housewife. Most children (59%) were still breastfed and in addition also given an average of 3.2 other meals per day containing 1.7 food groups. Children were sick for a total of 15 days during follow-up.

### 3.4. Cumulative Relapse Incidence

The cumulative incidence of relapse within the 6-month follow-up was 23.6% out of all that were enrolled and 26.1% when excluding dropouts (see Table 2). The cumulative incidence of relapse among children that had been admitted to treatment with a MUAC < 115 mm and/or edema was 23.0% when including all enrolled and 25.5% when excluding dropouts. The cumulative incidence of relapse to MUAC < 115 mm was low at 1.7% overall (Table S1). Only six children relapsed to MUAC < 115 mm or edema. The median time to relapse was 2.6 months (IQR 1.4; 4.0). The estimated ICC was 0.05 according to the 10 clusters included in the study.

### 3.5. Incidence Rate of Relapse

A child was on average seen 10 times during the 6-month follow-up period. The incidence rate of relapse was 4.8 per 100 child-months (Table 2). Relapse incidence rate was 4.8 [3.3; 7.1] per 100 child-months among children admitted with a MUAC < 115 mm and/or edema and 4.9 [3.9; 6.1] per 100 child-months among children admitted with a MUAC ≥ 115 mm to <125 mm.

### 3.6. Predictors of Relapse

Unadjusted and age- and sex-adjusted associations of different potential predictors with relapse are reported in Table 3. Relapse incidence was negatively associated with age (in months) with a hazard ratio (HR) [95%CI] of 0.94 [0.92; 0.97]. Relapse incidence was also negatively associated with WHZ, WAZ, and MUAC both at admission and at discharge from treatment: higher anthropometry predicted lower relapse incidence. The sex- and age-adjusted HRs (aHRs) were 0.63 [0.49; 0.80] for WHZ at discharge, 0.52 [0.40; 0.68] for WAZ at discharge, and 0.77 [0.69; 0.86] for MUAC at discharge. Children with a WHZ < −2 at discharge had a 1.67 [1.00; 2.79] higher hazard of relapse compared to those with a WHZ ≥ −2 at discharge (see Table S2). Children with a WAZ < −3 at discharge had a 3.09 [1.69; 5.62] higher hazard of relapse compared to children with a WAZ ≥ −3 at discharge. Children with a MUAC < 130 mm at discharge had a 2.90 [1.63; 5.14] higher hazard of relapse compared to children with a MUAC ≥130 mm at discharge. Relapse was not associated with sex or MUAC category at admission, nor with dietary habits. Relapse incidence was positively associated with the total number days with illness per month with an aHR of 7.57 [4.62; 12.42] and with the non-use of an improved water source (aHR = 1.93 [1.16; 3.20]) and with agriculture not being the main source of income (aHR = 1.76 [1.09; 2.84]). See Table 3 for associations of different continuous variables and Table S2 for categorical variables.

## 4. Discussion

We observed that 26% of children relapsed into MUAC < 125 mm after attaining a MUAC ≥ 125 mm for two consecutive visits following treatment with the simplified combined protocol. Cumulative relapse incidence was similar at 26% among children initially admitted to treatment with a MUAC < 115 mm and/or edema and those admitted with a MUAC ≥ 115 mm but <125 mm. Cumulative relapse incidence to MUAC < 115 mm and/or edema was very low at 1.7%. The incidence rate of relapse was 4.8 per 100 child months. Factors predicting relapse were lower age, lower anthropometric measures at admission to and at discharge from treatment, and higher number of illness episodes per follow-up month. Factors protecting from relapse included having a vaccination card, using an improved water source, having agriculture as the main source of income, and increases in caregiver workload.

### 4.1. Incidence of Relapse

Previous studies have reported relapse incidences ranging from 0% to 38% and, as concluded in a recent review on relapse to severe acute malnutrition (SAM), the challenge in any comparisons is the very different definitions used to declare relapse as well as the methods and length used to follow-up children [8]. The only previous study regularly following up children having initially been admitted to treatment with a MUAC < 125 mm and discharged with MUAC ≥ 125 mm reported that 6.3% of children had relapsed into MUAC < 125 mm in the first 3 months after discharge recovered [36]. This is much lower than the 26% cumulative relapse incidence observed in the current study. Part of this discrepancy can be attributed to the shorter follow-up time (3 months) in the study by Daures et al. (2021) [36] compared to the 6 months in this study. By using a longer follow-up, more children are expected to relapse. Moreover, as mentioned by Daures et al. (2021) [36], the incidence rate of relapse in their study might have been affected by concurrent programming for MAM, which did not occur in our study setting. It can also be that the OPTIMA protocol used in their study that involves a somewhat higher dose of RUTF during treatment reduces relapse after discharge. However, in a cross-sectional follow-up study of 850 children from the ComPAS RCT, no differences were found in the proportion of relapses between children after being treated with the simplified protocol versus standard protocol at 4 months after discharge from treatment [26]. Another study looking at the impact of a reduced dose of RUTF during treatment of SAM reported no difference in the proportion of relapse at 3 months post-recovery [37]. Generally, different study contexts, for example, in terms of the food security situation, morbidity patterns, and dietary and caregiving practices, would be expected to result in somewhat different relapse rates across contexts [10].

Another study conducted in Malawi looked at the relapse of children after treatment for MAM during a 12-month-long follow-up period with three follow-up time-points at 3, 6, and 12 months post-discharge [38]. They observed that 27% of all followed children relapsed into WHZ < −2 or MUAC < 125 mm during the 12 months. This is similar to the 26% observed in the current study. Most of the relapses (97%) in the study by Chang et al. (2013) [38] had occurred within the first 6 months.

We observed that 4.3% of children initially admitted to treatment with a MUAC < 115 mm relapsed to MUAC < 115 mm. A previous study in Nigeria reported a 26% cumulative incidence of relapse to MUAC < 115 mm among children initially admitted with MUAC < 115 mm and followed up fortnightly for 6 months [39]. Another study conducted in Malawi observed a 1.9% cumulative incidence of relapse to MUAC < 115 mm among children initially admitted to care with a MUAC < 115 mm and followed up fortnightly for 3 months [40]. The relatively low incidence of relapse to MUAC < 115 mm observed in the current study can mostly be attributed to the fact that children were followed up fortnightly and referred back to treatment as soon as a MUAC < 125 mm was detected. Thus, children undergoing wasting were probably caught before they had the time to regress to a MUAC < 115 mm.

Interestingly, children that had been admitted to treatment with a MUAC < 115 mm and/or oedema had a similar relapse rate to MUAC < 125 mm as those who had a MUAC between 115 and 124 mm at admission to treatment. However, relapse into MUAC < 115 mm seemed more common among children that had initially been admitted to treatment with a MUAC < 115 mm (4.3%) compared to the cumulative incidence among children with MUAC between 115 and 124 mm at admission to treatment (0.8%). Of note, only six children in total relapsed to MUAC < 115 mm, limiting the power of looking specifically at these cases.

Contrary to what was reported by Guesdon et al. (2021) [41], we did not find that the incidence of relapse decreased considerably in time; rather, it was relatively similar between the first 3 months of follow-up (5.3 per 100 child-months) and the latter 3 months (4.4 per 100 child months). It could have been that our children were somewhat better off at discharge, since being enrolled to our study required meeting the recovery criteria of MUAC ≥ 125 mm upon two consecutive visits and a discharge ratio of seven RUTF sachets was given to all children upon recovery. The total relapse incidence of 4.8 observed in the current study was also lower in general compared to the 7.2 reported by Guesdon et al. (2021) [41], which can also be attributed to the generally higher anthropometric indices at discharge from treatment in our study population.

### 4.2. Treatment Related Predictors of Relapse

Recently, a framework was proposed categorizing factors potentially related to relapse into those specifically related to the treatment and the sustainability of recovery and those that more globally predict malnutrition in a context [10].

In our study, the fact that we were studying children undergoing simplified treatment that uses only MUAC and oedema to admit and discharge children warranted looking specifically into anthropometry at discharge to identify whether children that do not reach WHZ ≥ −2 were more at risk of relapse. A previous study conducted in Nepal identified WHZ < −2 at discharge as the strongest anthropometric predictor of relapse into SAM [41]. In our study, while there seemed to be a trend (aHR 1.67, *p* = 0.052) towards higher risk among children having a WHZ < −2 at discharge, WHZ seemed a much less strong a predictor compared to WAZ. A WAZ < −3 at discharge from treatment carried a threefold hazard of relapse (aHR = 3.09, *p* < 0.001). The study in Nepal did not look at relapse according to WAZ [41]. Recent research has shown that WAZ is a more sensitive indicator than WHZ for capturing all children with increased risk of mortality due to anthropometric deficits [42,43]. A WAZ < −3 seems to capture children that have both wasting and stunting and who have a very high (12.6-fold) risk of mortality [44]. This suggests that WAZ could be a more useful indicator than WHZ to target, monitor, and treat children in order to decrease infant mortality and prevent relapse among children treated.

Children discharged with a MUAC < 130 mm had a near threefold hazard of relapse compared to those discharged with a MUAC of ≥130 mm. This invites the question on whether recovery criteria should be increased to 130 mm to decrease relapse post-recovery. To propose the most effective strategy for treatment, it would be important to judge both the sensitivity and specificity of the anthropometric measures to identify children that are at risk of mortality if not treated. For example, whether increasing discharge criteria to MUAC ≥ 130 mm actually prevents more mortality than adding another admission (and discharge) criteria such as WAZ < −3. Yet, solutions should also be judged against feasibility; in emergency contexts, MUAC is often the only feasible anthropometric measure easily adopted even by non-literate health workers [45].

We found that longer length of stay predicted relapse. It can be that children remaining longer in treatment suffered from some sub-clinical illnesses and were generally less healthy than those recovering faster, which would also translate to higher relapse post-discharge. In order to determine whether the treatment has been sufficient, it might be necessary to look into outcomes beyond anthropometry, such as immunological and nutritional biomarkers. Recent research has shown that lean tissue and micronutrient status is still sub-optimal at discharge after recovery from SAM [46,47,48,49]. There might be a need to reconsider what “recovery” means, and research studies looking at treatment response and relapse should incorporate biomarkers to enable more holistic understanding of children’s health and nutrition status at admission and discharge from treatment.

Finally, in our study context, children could be treated both at the formal health center level or at the community health sites, and we wanted to observe whether this could be a risk factor for relapse. There seemed to be no indication of a differential risk of relapse among children at the formal health care centers (aHR = 0.73, *p* = 0.192) compared to community health sites. This is a positive finding as it suggests that being discharged as recovered after treatment by a less qualified staff member does not put the child at higher risk of relapse post-treatment.

### 4.3. Contextual Predictors of Relapse

We analyzed a panel of factors that might not specifically predict relapse but may predict in general the occurrence of malnutrition in the study context as per the framework proposed by Shaefer et al. (2021) [10].

Total number of days with illness per month of follow-up was a strong predictor of relapse (aHR = 7.57, *p* < 0.001). Illness is considered as an independent causal factor leading to malnutrition both via decreased appetite as well as increased nutritional needs due to increased metabolism during illness and increased nutritional losses [50]. In the current study, if an illness was detected during a home visit, enumerators encouraged caregivers to bring the child to care. However, no follow-up was done to see if care was sought or obtained. Up to 96% of children had at least one illness episode during follow-up, but only 15% had sought care at least once from a formal healthcare structure. Preventing and limiting the duration of illness episodes seems critical for reducing relapse in this context. Encouraging caregivers to seek appropriate care when their child presents with any symptoms should be recommended.

Not using an improved water source seemed to predict relapse (aHR = 1.93, *p* = 0.012). This is in line with expectations, even though the evidence behind water sanitation and hygiene interventions in improving nutrition status is still inconclusive [51]. Up to 32% of the study population did not use an improved water source. Increasing access to safe water could reduce both malnutrition and relapse incidence.

Relying mainly on herding or trading activities as the main source of income seemed to predict relapse (aHR = 1.76, *p* = 0.020) as opposed to children from households relying on agriculture. Previous studies conducted in other regions of Mali have rather suggested that owning livestock protects from malnutrition [52]. The current study was conducted over a 10-month period, starting during the main harvest season and ending during the next agricultural lean season [53]. Thus, while households depending on agriculture could be expected to be better off at the harvest and immediate post-harvest season, the inverse would be expected towards the end of the study period during agricultural lean season.

We found that having a vaccination card available at enrolment was associated with lower relapse, while actually being up to date on vaccines was not. In a study in Burkina Faso, relapse was negatively associated with having been completely vaccinated [54]. Only 39% of the children included in the current study had a vaccination card available at enrolment, and of those, only 53% were up to date on their vaccines. We hypothesize that having a vaccine card available during treatment visits means these caregivers are more health conscious in general, possibly attending formal healthcare more regularly, as observed in a study in Senegal [55].

Increase in caregivers’ activities was associated with a decreased relapse rate. This was contrary to our expectation, whereby this increase in activities was expected to compete with caregiving duties and potentially increase the risk of relapse. In most cases, the increase in the workload was related to agricultural activities. However, controlling for agriculture as a main source of income did not attenuate the association. Neither did the workload increase translate to caregivers declaring higher income. It may be that attending field work meant that caregivers and potentially the entire household had better means to care for the child, explaining the lower relapse incidence.

Interestingly, we did not find that relapse incidence was associated with dietary patterns. This could suggest that malnutrition is more related to morbidity than dietary intake [56] in this context, as also suggested by the strong predictive value of illness episodes on relapse incidence. Defining malnutrition only on the basis of MUAC detects younger children than when also using the WHZ definition [57]. It has been shown that younger children are more prone to morbidity than older children [58]. It could be that in a context where children treated are older possibly due to using both MUAC and WHZ cut-offs, relapse would also be differently associated with dietary factors. It may also be that while dietary diversity and meal frequency were not associated with relapse, quantity of intake would be. However, we did not measure the quantity of foods consumed. Furthermore, dietary diversity reported by caregivers was extremely low among study children with an average of 1.7 groups consumed per day, speaking to a generally poor diet quality among study children. Post-discharge nutrient supplementation strategies, for example, using SQ-LNS that have been shown to reduce mortality [59], severe wasting [60], severe stunting [60], iron deficiency anemia [61], and developmental delay [62] among young children 6–24 months of age, could be tested to also reduce relapse and remaining micronutrient deficiencies, as well as improve longer-term health and nutrition status.

### 4.4. Recommendations, Strengths, and Limitations

Given the relatively high relapse (26%) into MUAC < 125 mm, three recommendations can be given. First, it may be relevant in this context to encourage the monitoring of children after discharge to catch and effectively treat the common illnesses children were presenting and that seemed to strongly predict relapse. Monitoring could be done via CHW or community health volunteer led home-visits or post-discharge follow-up visits to the health center. Second, given the extremely low diet diversity score, post-discharge supplementation strategies could be tested to decrease relapse. Though dietary patterns did not seem to predict relapse in this study, diet is an underlying cause of malnutrition [56], and supplementation may motivate caregivers to attend regular post-discharge follow-ups. Third, the important proportion of relapses observed in this study warrants discussion around the potential need to redefine the discharge criteria from malnutrition treatment. This study enrolled children who were discharged recovered following simplified treatment, thus having attained a MUAC ≥ 125 mm upon two consecutive visits. However, 16% of these children had a WHZ < −2 and 12% had a WAZ < −3 at discharge from treatment. Both WHZ and WAZ at discharge were strong predictors of relapse during follow-up, as was MUAC at discharge. It could be that a higher MUAC cut-off or using an additional anthropometric indicator is needed to ensure sustainable recovery. In a previous study, 100% of deaths in a treatment program could be captured with MUAC < 130 mm [57]. The strengths of this study include the use of fortnightly home visits by extensively trained study enumerators. The average number of 10 visits per participant during the 6-month follow-up period testifies to the regularity of the visits conducted and thus the completeness of data contributing to the precision of the relapse incidence estimation. Data collected included not only anthropometrics but also household characteristics enabling a more comprehensive analysis of potential risk factors of relapse.

The study also had limitations. First, some measures were incomplete for all children. For example, for children treated at CHW sites, no height measurements were taken. Imputation of height measures was used to maximize our ability to detect associations with relapse for anthropometric indexes such as HAZ or WHZ at admission or discharge. Second, we only enrolled children over a 5-month period, which does not allow us to look at the potential seasonal differences in relapse. However, as pointed out by Schaefer et al. (2021) [10], the seasonal effects are expected to be associated with malnutrition incidence in general and not specific to relapse incidence. Third, we did not power the study for looking specifically at predictors of relapse to SAM of children admitted initially with SAM. Our sample only included 114 children that were initially admitted to treatment with a MUAC < 115 mm and/or oedema. Their relapse incidence to MUAC < 125 mm was very similar to those admitted with a MUAC between 115 and 124 mm at admission to treatment. However, their relapse incidence to MUAC < 115 mm seemed somewhat higher than for those admitted with a MUAC between 115 and < 125 mm, but the small sample size did not allow for a conclusive interpretation. It remains to be understood whether children having suffered from SAM represent a specific group of children with higher risk of relapse and different predictive factors compared to children with MAM. Finally, although this study was observational by design, the lack of control group prevents us from understanding the role of the simplified treatment protocol, if any, in relapse.

## 5. Conclusions

In conclusion, we found a relatively high (26%) cumulative relapse incidence to MUAC < 125 mm among children treated successfully with a simplified combined protocol during a 6-month follow-up post-recovery. Monitoring children post-discharge should be warranted in this context to catch and treat illnesses early. Post-discharge supplementation strategies may support overall health and nutritional status and encourage caregivers to seek follow-up care. Finally, the discharge criteria may need to be reconsidered, either by expanding the MUAC criteria or adding WAZ, in order to allow for a more sustained recovery from malnutrition.

## Figures and Tables

**Table 1:** Descriptive characteristics of children enrolled in the relapse study.

	N Not Missing	Value
**1. Demographic and admission characteristics to malnutrition treatment**		
Male, % (n)	420	48.1 (202)
Age (months), median [IQR]	420	12.9 [10.0; 22.4]
WHZ, mean ± SD	270 *	−2.6 ± 1.2
WHZ < −3, % (n)	270 *	31.1 (84)
WAZ, mean ± SD	420	−2.9 ± 1.0
WAZ < −3, % (n)	420	45 (189)
HAZ, mean ± SD	270 *	−2.0 ± 1.5
HAZ < −3, % (n)	270 *	26.3 (71)
Admission category, % (n)	420	
MUAC < 115 mm and/or edema		26.7 (112)
MUAC ≥ 115 mm < 125 mm		73.3 (308)
Admission site, % (n)	420	
Treated at health center level		64.8 (272)
Treated at CHW site level		35.2 (148)
**2. Characteristics at discharge recovered from treatment**		
WHZ, mean ± SD	326 *	−1.2 ± 0.9
WHZ < −2, % (n)	326 *	16.0 (52)
WAZ, mean ± SD	413	−2.0 ± 0.9
WAZ < −3, % (n)	413	11.9 (49)
HAZ, mean ± SD	327 *	−2.1 ± 1.3
HAZ < −3, % (n)	327 *	26.3 (86)
MUAC (mm), mean ± SD	420	128.3 ± 2.6
MUAC < 130 mm, % (n)	420	68.6 (288)
Child’s vaccination card is available at enrolment, % (n)	420	38.8 (163)
Child’s vaccine status is up to date at enrolment, % (n)	163	53.4 (87)
Length of stay in treatment, median [IQR]	420	34 [23; 49]
**3. Socio-economic characteristics collected at first home visit**		
Household size, mean ± SD	413	20.9 ± 14.1
Number of children under 5 years of age in the household, mean ± SD	413	5.2 ± 3.7
Main source of income is from agriculture, % (n)	413	81.4 (336)
Caregiver has no other occupation than housewife duties, % (n)	413	73.6 (304)
Household uses an improved water source, % (n)	413	68.0 (281)
Household uses a product to treat drinking water, % (n)	413	17.4 (72)
Household uses a latrine, % (n)	413	54.2 (224)
Child is unclean, % (n)	415	27.5 (114)
Little or no hunger in household, % (n)	396	96.7 (383)
**4. Dietary habits collected at first home visit**		
Breastfeeding, % (n)	415	58.8 (244)
Number of food groups eaten, mean ± SD	415	1.7 ± 0.8
Frequency of feeding solids or semi-solids, mean ± SD	415	3.2 ± 1.2
**5. Morbidity during post-discharge follow-up**		
Total number of days sick during follow-up, mean ± SD	415	15.0 ± 10.2
Total number of days sick per month of follow-up, mean ± SD	415	3.5 ± 3.1
Child was sick at least once during follow-up, % (n)	415	95.9 (398)
Caregiver has sought formal care for an illness during follow-up in case child has been sick, % (n)	398	15.1 (60)
**6. Changes in the household level during follow-up**		
Income has decreased during follow-up, % (n)	402	17.7 (71)
Caregivers activities have increased during follow-up, % (n)	402	26.9 (108)
Hunger has increased during the follow-up, % (n)	402	3 (12)

* Height-related measures include missing values due to random omission of height measures during treatment and because no height measurements were taken by community health workers at the secondary sites. Abbreviations: FU, follow-up; HAZ, height-for-age z-score; HR, hazard ratio; IQR, inter quartile range; MDD, minimum dietary diversity; MDF, minimum dietary frequency; MUAC, mid-upper arm circumference; SD, standard deviation; U5, under 5 years of age; WAZ, weight-for-age z-score; WHZ, weight-for-height z-score.

**Table 2:** Cumulative incidence and incidence rate of relapse to MUAC < 125 mm or edema.

	MUAC and Edema Status at Admission to Treatment					
	MUAC < 125 mm and/or Edema		MUAC < 115 mm and/or Edema		MUAC 115 mm to MUAC < 125 mm	
	n/N	% [95%CI]	n/N	% [95%CI]	n/N	% [95%CI]
**Cumulative incidence over the 6-month follow-up**						
All children enrolled	99/420	23.6 [19.6; 27.9]	26/113	23.0 [15.6; 31.9]	73/307	23.8 [19.1; 28.9]
Non-drop outs	99/380	26.1 [21.7; 30.8]	26/102	25.5 [17.4; 35.1]	73/278	26.3 [21.2; 31.8]
**Incidence rates** **(per 100 child-months)**						
First 3 months	59/1136	5.2 [4.0; 6.7]	19/305	6.2 [4.0; 9.8]	40/830	4.8 [3.5; 6.6]
Latter 3 months	40/909	4.4 [3.2; 6.0]	7/235	3.0 [1.4; 6.3]	33/674	4.9 [3.5; 6.9]
Full 6 months	99/2045	4.8 [4.0; 5.9]	26/540	4.8 [3.3; 7.1]	73/1505	4.9 [3.9; 6.1]

**Table 3:** Associations of different characteristics (as continuous variables) with the 6-month incidence of relapse among children discharged as recovered following treatment with the simplified protocol in 10 randomly selected health areas of the Nara district in Mali.

	Unadjusted		Sex and Age Adjusted	
	HR [95%CI]	*p*-Value	HR [95%CI]	*p*-Value
**1. Demographic and admission characteristics to malnutrition treatment**				
Age (months)	0.94 [0.91; 0.97]	<0.001	0.65 [0.51; 0.84] *	0.001
WHZ	0.89 [0.75; 1.06]	0.203	0.82 [0.69; 0.99]	0.040
WAZ	0.91 [0.75; 1.12]	0.37	0.75 [0.60; 0.93]	0.009
HAZ	1.05 [0.91; 1.20]	0.51	0.94 [0.80; 1.09]	0.39
MUAC (mm)	0.97 [0.94; 1.00]	0.081	0.97 [0.93; 1.00]	0.072
**2. Characteristics at discharge** **recovered from treatment**				
WHZ	0.71 [0.56; 0.90]	0.005	0.63 [0.49; 0.80]	<0.001
WAZ	0.75 [0.59; 0.94]	0.014	0.52 [0.40; 0.68]	<0.001
HAZ	0.99 [0.85; 1.15]	0.87	0.86 [0.73; 1.02]	0.083
MUAC (mm)	0.75 [0.67; 0.84]	<0.001	0.77 [0.69; 0.86]	<0.001
Length of stay (d)	1.01 [1.01; 1.02]	<0.001	1.01 [1.01; 1.02]	0.001
**3. Socio-economic characteristics**				
Household size	0.99 [0.97; 1.00]	0.14	0.99 [0.97; 1.00]	0.13
Number of children U5 in the household	0.96 [0.90; 1.02]	0.22	0.96 [0.90; 1.02]	0.19
**4. Dietary habits collected at first home visit**				
Number of food groups eaten	0.77 [0.57; 1.05]	0.103	0.84 [0.62; 1.15]	0.28
Frequency of feeding solids or semi-solids	0.81 [0.68; 0.96]	0.013	0.90 [0.75; 1.08]	0.26
**5. Morbidity during post-discharge FU**				
Total number of days sick during FU	0.99 [0.97; 1.01]	0.274	0.98 [0.96; 1.00]	0.09
Total number of days sick per month of FU	1.31 [1.25; 1.36]	<0.001	1.30 [1.24; 1.35]	<0.001

Abbreviations: FU, follow-up; HAZ, height-for-age z-score; HR, hazard ratio; MDD, minimum dietary diversity; MDF, minimum dietary frequency; MUAC, mid-upper arm circumference; U5, under 5 years of age; WAZ, weight-for-age z-score; WHZ, weight-for-height z-score. * only adjusted for sex.

## Data Availability

Data may be accessed and downloaded at https://doi.org/10.5281/zenodo.7938179 (accessed on 16 May 2023).

## Supplementary Materials

The following supporting information can be downloaded at https://www.mdpi.com/article/10.3390/nu15112636/s1, Table S1: Cumulative incidence and incidence rate of relapse to MUAC < 115 mm and/or edema. Table S2: Associations of different characteristics (as categorical variables) with the 6-month incidence of relapse among children discharged as recovered following treatment with the simplified protocol in 10 randomly selected health areas of the Nara district in Mali.
