# Supplementary material for: Post-Recovery Relapse of Children Treated with a Simplified, Combined Nutrition Treatment Protocol in Mali: A Prospective Cohort Study
Source: Nutrients. 2023 Jun 5;15(11):2636. doi: 10.3390/nu15112636 (PMC10255596; doi:10.3390/nu15112636)
Supplement: Supplementary file 1 [file nutrients-15-02636-s001.zip › nutrients-2427749-supplementary.pdf]

Supplementary Materials

Post-Recovery Relapse of Children Treated with a Simplified, Combined Nutrition Treatment Protocol in Mali: A Prospective Cohort Study

Table S1. Cumulative incidence and incidence rate of relapse to MUAC <115 mm and/or edema

	MUAC and edema status at admission to treatment					
	MUAC <125 mm and/or edema		MUAC <115 mm and/or edema		MUAC 115 mm to MUAC <125 mm	
	**n/N**	**% [95%CI]**	**n/N**	**% [95%CI]**	**n/N**	**% [95%CI]**
**Cumulative incidence over the 6 months follow-up**						
All children enrolled	6 / 389	1.5 [0.6 ; 3.3]	4 / 103	3.9 [1.1 ; 9.6]	2 / 286	0.7 [0.1 ; 2.5]
Non-drop outs	6 / 355	1.7 [0.6 ; 3.6]	4 / 94	4.3 [1.2 ; 10.5]	2 / 261	0.8 [0.1 ; 2.7]
**Incidence rates (per 100 child-months)**						
First 3 months	5 / 1136	0.4 [0.2 ; 1.1]	3 / 305	1.0 [0.3 ; 3.0]	2 / 830	0.2 [0.1 ; 1.0]
Latter 3 months	1 / 909	0.1 [0.0 ; 0.8]	7 / 235	0.4 [0.1 ; 3.0]	0 / 674	
Full 6 months	6 / 2045	0.3 [0.1 ; 0.7]	4 / 540	0.7 [0.3 ; 2.0]	2 / 1505	0.1 [0.0 ; 0.5]

Table S2. Associations of different characteristics (as categorical variables) with the 6-month incidence of relapse among children discharged as recovered following treatment with the simplified protocol in 10 randomly selected health areas of the Nara district in Mali

	Relapsed				Unadjusted		Sex and age adjusted	
	Yes, n	No, n	Proportion, %	FU time (months)	HR [95%CI]	p-value	HR [95%CI]	p-value
**1. Demographic and admission characteristics to malnutrition treatment**								
**Sex**								
Female	51	167	23.4%	4.8	Ref		Ref	
Male	48	154	23.8%	4.9	1.01 [0.68 ; 1.50]	0.969	0.98 [0.66 ; 1.46]	0.911
**Age category**								
≥24mo	6	56	9.7%	5.2	Ref		Ref	
<24m	93	265	26.0%	4.8	2.85 [1.24 ; 6.52]	0.013	1.20 [0.43 ; 3.37]	0.731
**WAZ category**								
≥-3	54	177	23.4%	4.8	Ref		Ref	
<-3	45	144	23.8%	4.9	1.04 [0.69 ; 1.56]	0.850	1.41 [0.92 ; 2.17]	0.118
**WHZ category**								
≥-3	45	141	24.2%	4.7	Ref		Ref	
<-3	24	60	28.6%	4.6	1.18 [0.73 ; 1.91]	0.497	1.35 [0.83 ; 2.21]	0.228
**HAZ category**								
≥-3	51	148	25.6%	4.7	Ref		Ref	
<-3	18	53	25.4%	4.7	1.10 [0.69 ; 1.75]	0.688	1.49 [0.92 ; 2.42]	0.106
**MUAC category**								
≥115 <125mm	73	235	23.7%	4.9	Ref		Ref	
<115mm	26	86	23.2%	4.8	1.14 [0.72 ; 1.82]	0.568	1.17 [0.74 ; 1.85]	0.511
**Treatment level**								
health center	69	203	25%	4.7	Ref		Ref	
community health site	30	118	20%	5.2	0.67 [0.42 ; 1.06]	0.090	0.73 [0.46 ; 1.17]	0.192
**2. Characteristics at discharge recovered from treatment**								
**WAZ category**								

≥-3	82	282	22.5%	4.9	Ref		Ref	
<-3	16	33	32.7%	4.4	1.70 [0.99 ; 2.94]	0.056	3.09 [1.69 ; 5.62]	<0.001
**WHZ category**								
≥-2	66	208	24.1%	4.8	Ref		Ref	
<-2	16	36	30.8%	4.5	1.39 [0.84 ; 2.29]	0.199	1.67 [1.00 ; 2.79]	0.052
**HAZ category**								
≥-3	59	182	24.5%	4.8	Ref		Ref	
<-3	23	63	26.7%	4.7	1.28 [0.83 ; 1.99]	0.26	1.76 [1.11 ; 2.78]	0.016
**MUAC category**								
≥130mm	14	118	10.6%	5.3	Ref		Ref	
<130mm	85	203	29.5%	4.7	3.21 [1.82 ; 5.68]	<0.001	2.90 [1.63 ; 5.14]	<0.001
**Availability of a vaccination card at enrolment**								
Yes	34	129	20.9%	5.0	Ref		Ref	
No	65	192	25.3%	4.8	1.25 [0.81 ; 1.93]	0.31	1.57 [1.01 ; 2.45]	0.047
**Child's vaccine status is up to date at enrolment**								
Yes	20	67	23.0%	4.7	Ref		Ref	
No	14	62	18.4%	5.4	0.69 [0.32 ; 1.46]	0.33	0.73 [0.34 ; 1.56]	0.42
**Length of stay above median (=39d)**								
No	39	171	18.6%	5.0	Ref		Ref	
Yes	60	150	28.6%	4.7	1.86 [1.22 ; 2.82]	0.004	1.83 [1.21 ; 2.78]	0.005
**3. Socio-economic characteristics collected at first home visit**								
**Larger than average (n=21) household size**								
No	30	127	19.1%	5.0	Ref		Ref	
Yes	69	194	26.2%	4.8	1.52 [0.96 ; 2.41]	0.072	1.49 [0.94 ; 2.37]	0.089
**More than 5 children U5 in the household**								
No	31	120	20.5%	5.0	Ref		Ref	
Yes	68	201	25.3%	4.8	1.25 [0.79 ; 1.97]	0.34	1.33 [0.85 ; 2.10]	0.22

**Main source of income is from agriculture**								
Yes	71	265	21.1%	5.1	Ref		Ref	
No	26	51	33.8%	4.4	1.84 [1.14 ; 2.95]	0.012	1.76 [1.09 ; 2.84]	0.020
**Caregiver has other occupation that house-wife duties**								
Yes	22	87	20.2%	5.0	Ref		Ref	
No	75	229	24.7%	4.9	0.98 [0.55 ; 1.74]	0.94	1.03 [0.58 ; 1.83]	0.93
**Household uses an improved water source**								
Yes	61	220	21.7%	5.0	Ref		Ref	
No	36	96	27.3%	4.8	1.96 [1.14 ; 3.36]	0.015	1.93 [1.16 ; 3.20]	0.012
**Household uses a product to treat drinking water**								
Yes	84	257	24.6%	5.0	Ref		Ref	
No	13	59	18.1%	4.9	1.39 [0.76 ; 2.57]	0.29	1.58 [0.85 ; 2.91]	0.146
**Household uses a latrine**								
Yes	59	165	26.3%	4.8	Ref		Ref	
No	38	151	20.1%	5.1	0.76 [0.49 ; 1.20]	0.24	0.80 [0.51 ; 1.26]	0.34
**Child appears unclean**								
No	74	227	24.6%	4.9	Ref		Ref	
Yes	25	89	21.9%	4.9	1.00 [0.60 ; 1.67]	0.99	1.23 [0.74 ; 2.05]	0.43
**Little or no hunger in household**								
Yes	83	300	21.7%	5.1	Ref		Ref	
No	6	7	46.2%	4.3	2.47 [1.05 ; 5.77]	0.037	2.22 [0.95 ; 5.18]	0.066
**4. Dietary habits collected at first home visit**								
**Breastfeeding**								
No	29	142	28.7%	5.1	Ref		Ref	
Yes	70	174	17.0%	4.8	1.73 [1.12 ; 2.68]	0.014	0.92 [0.52 ; 1.64]	0.78
**Attained MDD**								
Yes	0	4	0%	6.0	Ref		Ref	
No	83	217	28.0%	4.8	NA		NA	
**Attained MDF**								

Yes	43	92	31.9%	4.7	Ref		Ref	
No	40	129	23.7%	4.9	0.70 [0.44 ; 1.12]	0.136	0.80 [0.49 ; 1.30]	0.37
**5. Morbidity during follow-up**								
**Above average total number of sick days during follow-up**								
No (=<15)	54	183	22.8%	4.6	Ref		Ref	
Yes (>15)	45	138	24.6%	5.3	0.85 [0.56 ; 1.30]	0.46	0.76 [0.50 ; 1.16]	0.20
**Above average (>3.5) number of sick days per month of follow-up**								
No (=<3.5)	23	243	8.7%	5.3	Ref		Ref	
Yes (>3.5)	76	78	49.4%	4.0	8.38 [5.14 ; 13.66]	<0.001	7.57 [4.62 ; 12.42]	<0.001
**Child sick at least once during follow-up**								
No	96	302	24.1%	5.0	Ref		Ref	
Yes	3	14	17.7%	2.7	1.30 [0.40 ; 4.18]	0.66	1.28 [0.40 ; 4.16]	0.68
**Caregiver sought formal care when the child was sick**								
Yes	75	263	22.2%	5.0	Ref		Ref	
No	21	39	35.0%	5.0	1.29 [0.76 ; 2.19]	0.35	1.16 [0.68 ; 1.95]	0.59
**6. Changes in the household level during follow-up**								
**Income has decreased during follow-up**								
No	76	255	23.0%	5.0	Ref		Ref	
Yes	16	55	22.5%	5.4	0.65 [0.35 ; 1.20]	0.167	0.69 [0.37 ; 1.28]	0.24
**Increase in caregivers activities during follow-up**								
No	71	223	24.2%	4.9	Ref		Ref	
Yes	21	87	19.4%	5.6	0.33 [0.18 ; 0.58]	<0.001	0.32 [0.18 ; 0.57]	<0.001
**Increase in hunger during the follow-up**								
No	89	301	22.8%	5.1	Ref		Ref	
Yes	3	9	25.0%	5.2	2.78 [0.66 ; 11.78]	0.164	3.60 [0.82 ; 15.72]	0.089

* only adjusted for sex

Abbreviations: FU, follow-up; HAZ, height-for-age z-score; HR, hazard ratio; MDD, minimum dietary diversity; MDF, minimum dietary frequency; MUAC, mid-upper arm circumference; U5, under 5 years of age; WAZ, weight-for-age z-score; WHZ, weight-for-height z-score.
